# Hepatoprotective and Antioxidant Effect of *Mangifera Indica* Leaf Extracts against Mercuric Chloride-induced Liver Toxicity in Mice

**DOI:** 10.5005/jp-journals-10018-1091

**Published:** 2014-01-22

**Authors:** Muthupillai Karuppanan, Manigandan Krishnan, Pavankumar Padarthi, Elangovan Namasivayam

**Affiliations:** 1 Department of Biotechnology, Periyar University, Salem, Tamil Nadu, India; 2 Department of Biotechnology, Periyar University, Salem, Tamil Nadu, India; 3 Department of Biotechnology, Periyar University, Salem, Tamil Nadu, India; 4 Department of Biotechnology, Periyar University, Salem, Tamil Nadu, India

**Keywords:** *Mangifera indica*, Mercuric chloride, Free radicals, Liver enzymes, Antioxidant.

## Abstract

**Background:**

To explore the antioxidant and hepatoprotective effect of ethanolic Mangifera indica (EMI) and methanolic *Mangifera indica* (MMI) leaf extracts in mercuric chloride (HgCl_2_) induced toxicity in Swiss albino mice.

**Materials and methods:**

Toxicity in mice was induced with HgCl_2_ (5.0 mg/kg, i.p.), followed by oral intervention with EMI and MMI extracts (25 mg and 50 mg/kg. body wt.) for 30 days.

**Results and discussion:**

The extent of liver damage was assessed from the extents of histopathological, morphological, antioxidant and liver enzymes. Mercuric chloride-induced mice showed an increased cellular damage whereas leaf extracts of EMI and MMI-treated mice showed recovery of damaged hepatocytes. Mercuric chloride intoxicated mice exhibited a significant (p < 0.05) elevation in the liver enzymes (Aspartate amino transferase and Alanine amino transferase) and gradual decline in the cellular radical scavenging enzyme levels (Catalase, Glutathione-s-transferase and Glutathione peroxidase. The combined treatment with EMI and MMI leaf extracts significantly (p < 0.05) reversed these parameters. However, the effects of MMI leaf extract (50 mg/kg) were superior to those of EMI- treated mice possibly due to its potent radical scavenging property. These results suggest that oral supplementation of *Mangifera indica* extract remarkably reduces hepatotoxicity in mice possibly through its antioxidant potentials.

**How to cite this article:** Karuppanan M, Krishnan M, Padarthi P, Namasivayam E. Hepatoprotec-tive and Antioxidant Effect of *Mangifera Indica* Leaf Extracts against Mercuric Chloride-induced Liver Toxicity in Mice. Euroasian J Hepato-Gastroenterol 2014;4(1):18-24.

## INTRODUCTION

Mercury (Hg), a toxic environmental pollutant,^1^ elicits both acute and chronic liver injury.^2^ Exposure to inorganic mercury can form a complex with thiol group (R-SH) and firmly causes tissue damage, alterations in transcriptional factors and other pathological disorders.^3^ Liver is the prime target for mercury toxicity due to its metabolic activities. Earlier reports suggest that Hg^2+^ and Cl^-^ are formed as a result of metabolic activation of HgCl_2_, which can promote lipid peroxidation, tissue injury and DNA damage due to production of reactive oxygen species (ROS),^4^ along with significant decrease of antioxidant scavenging enzymes such as catalase alase, glutathione-s-transferase, and glutathione peroxidise. Earlier studies predict that antioxidants play a prime role to counteract the adverse effects of metabolic free radical products.^5^ Hence, plant-based remedies may provide a protection against mercury-induced toxicity with fewer side effects. Herbal plant represents a quest of unknown metabolites; analysis of those active metabolites is in need for the prevention and treatment of several chronic diseases.

*Mangifera indica* (MI) Linn is one of the prevalent tropical plants widely used in Indian ayurvedic medicine.^6^ MI is traditionally used for the treatment of diabetes,^7^ hepatoprot-ecitve,^8^ radioprotective,^9^ cell migration activity,^10^ antidiarr-heal,^11^ anticancer activity,^12^ and antimicrobial activity.^13^ However, heavy metal toxicity of MI leaf extract has not yet documented. In this study, we investigated the possible hepatoprotective efficacy of EMI and MMI leaf extracts against mercuric chloride-induced oxidative stress in mice.

## MATERIALS AND METHODS

### Chemicals

Mercuric chloride, L-aspartic acid, L-alanine, 1, 1-Diphenyl-2-picrylhydrazyl (DPPH), 2, 4-dinitrophenylhydrazine (DNPH), 5, 5 dithiobis (2-nitrobenzoic acid) (DTNB), reduced glutathione, 1-chloro-2, 4-dinitrobenzene (CDNB), sodium azide, trichloroacetic acid (TCA), O-dianisidine, hydrogen peroxide (H_2_O_2_), ethylene diamine tetraacetic acid (EDTA), hemotoxylin and eosin were purchased from Sigma Chemical Co. St. Louis, MO, USA. All chemicals are of analytical grade.

### Animals

Adult male Swiss albino mice (4-6 weeks, weighing 2225 gm) were purchased and housed at Central Animal Facility, Periyar University, Salem, Tamil Nadu, India under controlled conditions (12 hours day-night cycle), temperature (25 ± 2°C) and relative humidity (45 ± 5%). Mice were caged in polypropylene cages and fed with a commercial pelleted food and tap water *ad libitum.* The study was conducted after obtaining institutional animal ethical committee clearance (1085/ac/07/PU-IAEC 2011/01).

### Plant Material

MI Linn leaves were collected from in and around Salem district. The plant was identified and a voucher specimen (PU/BT/Mangz/era *indica.* Linn/ S. No. 011/2010) was stored in the herbarium of Department of Biotechnology, Periyar University. The leaves were washed, shade dried, powdered and extraction was carried out using methanol and ethanol in soxhlet apparatus for 6 hours. The extract was concentrated to dryness by using rotary evaporator at 40 to 50°C under reduced pressure.

**Table 1:** **Table 1:** Treatment schedule

*Groups*		*Route of administration*	
Group I-Control		0.9% (w/v) Saline orally for 30 days	
Group II-HgCl_2_ treatment		Intraperitoneal induction of HgCl_2_ (5.0 mg/kg b. wt. in 0.9% saline) for 30 days	
Group III-HgCl_2_ + 25 mg/kg EMI extract treatment		HgCl_2_ as prescribed in group II followed by aqueous suspension of EMI extract, orally up to 30 days	
Group IV-HgCl_2_ + 50 mg/kg EMI extract treatment		HgCl_2_ as stipulated in group II followed by aqueous suspension of EMI extract, orally for the entire period	
Group V-HgCl_2_ + 25 mg/kg MMI extract treatment		HgCl_2_ as mentioned in group II followed by oral administration of aqueous MMI extracts for 30 days	
Group VI-HgCl_2_ + 50 mg/kg MMI extract treatment		HgCl_2_ as stated in group II followed by oral administration of aqueous MMI extracts for 30 days	

### Evaluation of Plant Radical scavenging Activity

The ability of MI leaf extracts to scavenge 1, 1-Diphenyl-2-picrylhydrazyl (DPPH) radical was assayed by the method of Liyana-Pathiranan.^14^ The percent (%) inhibition was calculated by the following equation: DPPH radical (%) = [(Abs - control- Abs_sample_)/(Abs_control_] × 100.

### Experimental Design

A total of 36 mice were divided into six experimental groups (n = 6) were shown in Table 1. The experiment was terminated at the end of 31st day and all the animals were sacrificed under anesthesia. Liver tissues were removed, rinsed in ice cold saline and immersed in 10% (v/v) formalin for histological examination and the rest of the liver tissues was homogenized with 10% (w/v) of ice cold potassium phosphate buffer (pH 7.4) and centrifuged at 12,000 gm for 20 minutes at 4°C. The supernatant was stored at -20°C for biochemical analysis.

### Biochemical Analysis

All the enzyme levels in tissue homogenate were measured spectrometrically (Systronics-2203, India Pvt. Ltd. The liver enzymes aspartate amino transferase (EC 2.6.1.1) and alanine amino transferase (EC 2.6.1.2) in tissue homogenate were assessed by the method of Bergmeyer and Bernt.^15^ The activities of both enzymes were expressed in U/min/ mg of protein. The protein concentration was measured by the method of Lowry et al.^16^ The antioxidant enzymes glutathione peroxidase (EC 1.11.1.9) catalyzed the oxidation of NADPH coupled with reduced glutathione at 420 nm. The enzyme activity was expressed as n moles of glutathione oxidized/min/ml/ of enzyme,^17^ glutathione S-transferase (EC 2.5.1.18) was performed as described by Habig et al^18^ is using 1-chloro-2,4-dinitrobenzene as substrate and catalase (EC 2.3.1.28) was measured by decrease in H_2_O_2_ reduction at 620 nm. The enzyme activity was expressed as n moles of H_2_O_2_ consumed/units/mg of protein.^19^

**Fig. 1:**
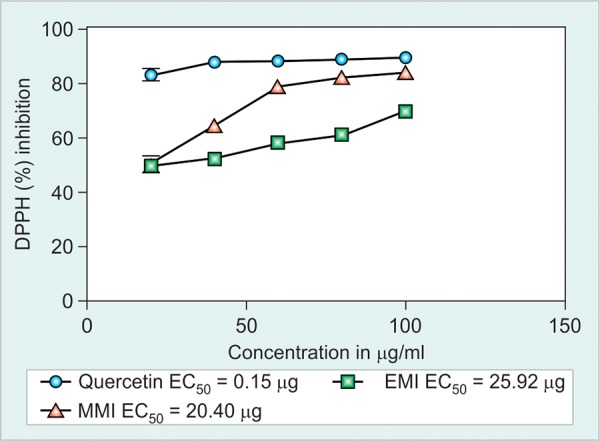
DPPH radical scavenging activity of ethanolic and methanolic *Mangifera indica* plant extracts compared with quercetin. The percentage inhibition was plotted against concentration of the sample and the IC_50_ values of both extract are expressed in ug/ml

### Histological Studies

A segment of the liver tissues was fixed in 10% formalin solution for 24 to 48 hours and dehydrated in descending alcohol concentration for 12 hours. Tissues were embedded in paraffin wax for 2 hours, sectioned in microtome at 6.0 urn size and then deparaffinized under xylene for 2 hours. The sections were stained with hemotoxylin and eosin and the slides were viewed under light microscope [Magnus MLXi, Olympus, India Pvt. Ltd] for histological changes.

## STATISTICAL ANALYSIS

All statistical analysis was conducted using one-way analysis of variance with Dunnett’s posttest using SPSS version 16.0. The value expressed as Mean ± SEM, p-value < 0.05 was considered as statistically significant.

**Figs 2A and B:**
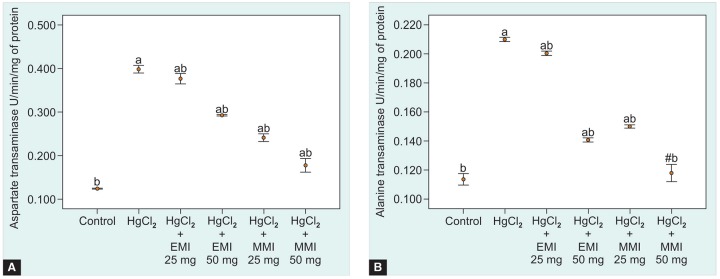
Effect of *Mangifera indica* leaf extracts on liver marker enzymes (A) Aspartate transaminase and (B) Alanine transaminase levels in tissue of control and experimental mice. Values are expressed as mean ± SEM for six mice in each group, Dunnett’s, values are statistically significant at ^a^p < 0.05 *vs* control group; ^b^p < 0.05 *vs* HgCl_2_ group and "nonsignificant *vs* control group

## RESULTS

### Biochemical Observations

**Figs 3A to C:**
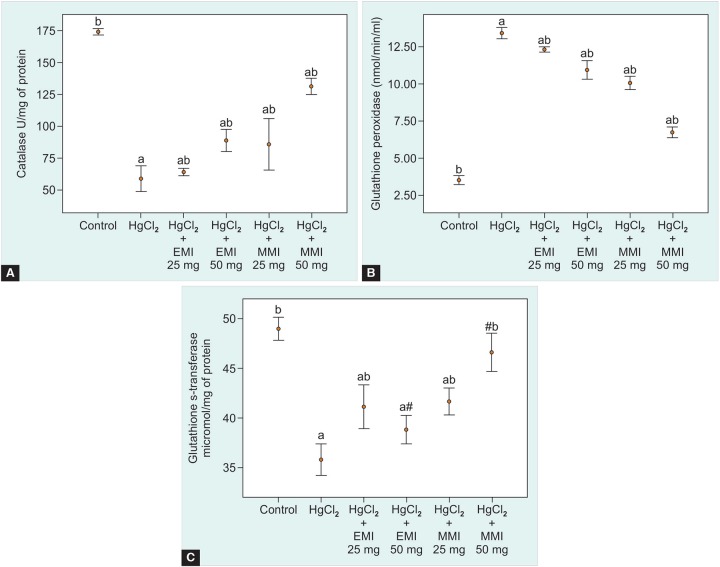
Dose-dependent effect of both ethanolic and methanolic fractions of *Mangifera indica* leaf extracts on (A) Catalase, (B) Glutathione peroxidase and (C) Glutathione s-transferase levels against HgCl_2_-induced hepatotoxicity in mice. Values are expressed as mean ± SEM for six mice in each group, values are statistically significant at ^a^p < 0.05 as compared with control group, ^b^p < 0.05 *vs* as compared with HgCl_2_ group and #nonsignificant *vs* control and HgCl_2_ groups

The radical scavenging activity of ethanol and methanol MI extracts, compared with standard was shown in Figure 1. The methanol extract produced 50% of inhibition activity (IC_50_) at 20.40 ug/ml concentration, while the ethanol extract produced 50% radical scavenging inhibition at 25.92 ug/ml concentration. This result suggests that methanol extract exhibits significant EC_50_ range at minimum concentration than ethanol extract. All the animals were survived at the entire study period. The average body and liver weight in all groups were shown in Table 2. Mercuric chloride treated group shows a significant (p < 0.01) decline in mean of final body and liver weight when compared with control group. Administration of plant extracts significantly (p < 0.01) recovered the mean of final body and liver weight, when compared to the intoxicated group. The activities of aspartate transaminase, alanine transaminase, glutathione peroxidase, glutathione s-transferase and catalase levels in liver of control and experimental groups were shown in the Figures 2 and 3. A significant elevation (p < 0.05) in liver marker enzymes aspartate transaminase and alanine transaminase levels was observed in HgCl_2_ alone intoxicated mice with respect to control. But, treatment with EMI and MMI leaf extracts with HgCl_2_ elicits a significant decline (p < 0.05) in tissue aspartate transaminase and alanine transaminase levels with respect to HgCl_2_ mice (see Figs 2 and 3). However, MMI extract possesses maximum quenching effect beyond the homeostasis levels.

The antioxidant enzyme (catalase, glutathione peroxi-dase and glutathione s-transferase) levels in hepatic tissue homogenate of mice in control and experimental groups were shown in the Figures 3A to C. Glutathione s-transferase, a cytosolic antioxidant belongs to an isozyme family, involved in scavenging the cellular reactive oxygen species (ROS). The enzyme level was significantly (p < 0.05) reduced in HgCl_2_ treated mice with respect to control mice. When compared to HgCl_2_ group, a significant (p < 0.05) elevation in glutathione s-transferase was observed in MI extract administered groups (Fig. 3C). The levels of glutathione peroxidase and catalase are involved in eliminating the proformed H_2_O_2_ and superoxide anion. When compared with the control group, HgCl_2_-treated group caused significant (p < 0.05) reduction in glutathione peroxidase and catalase levels. However, EMI and MMI leaf extracts-treated group showed a significant variation in these enzyme levels (Figs 3A and B).

**Table 2:** **Table 2:** Effect of EMI and MMI leaf extracts on body weight, liver weight and relative liver weight of mice

*Parameters*		*Control*		*5.0 mg HgCl_2_*		*EMI + HgCl_2_*				*MMI + HgCl_2_*			
						*25 mg + 5.0 mg*		*50 mg + 5.0 mg*		*25 mg + 5.0 mg*		*50 mg + 5.0 mg*	
Day 1^st^ body weight (g)		22.5 ± 0.54		22.83 ± 0.75		22.83 ± 0.75		23 ± 0.89		22.66 ± 0.81		22.83 ± 0.7528	
Day 15^th^ body weight (g)		23.8 ± 0.60		22.33 ± 0.60		22.26 ± 0.65		22.18 ± 0.51		22.15 ± 0.32		23.16 ± 0.71	
Day 30^th^ body weight (g)		24.5 ± 0.37^b^		20.91 ± 0.44^a^		22.4 ± 0.54^ab^		22.46 ± 0.26*^b^		22.46 ± 0.15^ab^		22.67 ± 0.24#^b^	
Liver weight (g)		1.38 ± 0.05^d^		1.24 ± 0.02^c^		1.3 ± 0.01^c^*		1.31 ± 0.02^cd^		1.317 ± 0.02^cd^		1.33 ± 0.01#^d^	
Relative liver weight (g/100 g body weight)		5.69 ± 0.16*		5.93 ± 0.19		5.77 ± 0.17		5.77 ± 0.08		5.52 ± 0.07		5.73 ± 0.08	

Data are analyzed by one-way analysis of variance followed by Dunnett’s test for six mice in each group. ^a^ ^c^ p < 0.01 and *p < 0.05 as compared with control group; #nonsignificant *vs* control group; ^b^ ^d^ p < 0.01 as compared with HgCl_2_ alone-induced group

**Figs 4A to F:**
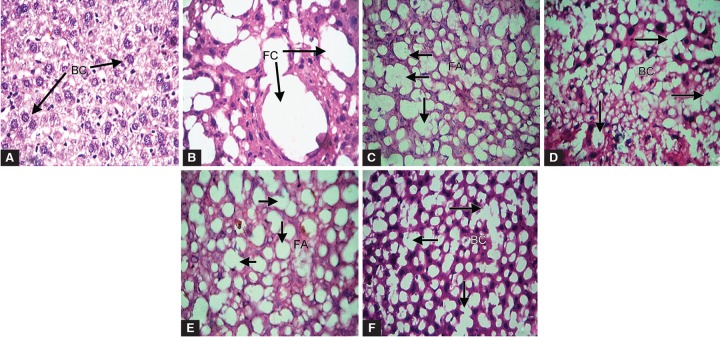
Microscopic evaluation of tissue morphology of HgCl_2_-induced Hepatotoxicity in mice. (A) Saline control-treated group exhibits normal hepatocyte; (B) HgCl_2_ (5.0 mg/kg) induced toxicity shows elevation of abnormal fat cyst formation; (C) EMI (25 mg/kg) combined with HgCl_2_ exhibits recovery of normal hepatocyte; (D) EMI (50 mg/kg) induced with HgCl_2_ shows significant recovery of normal hepatocyte; (E) HgCl_2_ treated with MMI (25 mg/kg) elicits recovery of liver cells; (F) MMI (50 mg/kg) administered with HgCl_2_ shows significant recovery of normal hepatocyte with reduced formation of fat cyst formation. (BC- Bi-nucleated cells, FC- fat cyst formation and FA- fat accumulation)

### Histopathological Findings

Histological changes in liver sections of control and experimental mice were shown in Figures 4A to F. The liver section of control mice displayed normal hepatocyte with negative signs of cellular abnormality (Fig. 4A). HgCl_2_ alone intoxicated mice (5.0 mg/kg) shows conventional degeneration of cellular necrosis, massive inflammation and fat cyst formation (Fig. 4B). When compared with mercuric chloride alone intoxicated groups, the liver sections of EMI and MMI leaf (25 mg and 50 mg/kg) extracts exhibited revival of normal cells upon necrotic damage in liver cells (Figs 4C and D). However, significant recovery of liver cell damage was observed in HgCl_2_ + MMI (50 mg/kg) extract-treated mice (Figs 4E and F) as compared with the HgCl_2_ + EMI leaf extract (50 mg/kg).

## DISCUSSION

Hepatocytes not only act as a prime target in eliminating the exogenous toxic substances by process of detoxification, also provides an excellent biomarker for diagnosis. Our investigation outlines the hepatoprotective effect of EMI and MMI leaf extract through its radical scavenging property against HgCl_2_-induced intoxication in hepatocyte of mice. Toxicity with HgCl_2_ can amend the biochemical changes through oxidative stress mediated cell injury by lipid peroxidation and also inactivate the cellular radical scavenging enzymes in liver such as catalase, glutathione peroxidase and glutathione s-transferase. Usually, antioxidant enzymes have the affinity to act as a primary scavenging intermediate of ROS. Hence, an increased level of ROS induces oxida-tive stress, resulting in the disturbance of prooxidant and simultaneously decreased the antioxidant enzyme levels due to over utilization to scavenge the products of ROS at the site of cell damage.^20^

In this study, we observed that the administration of HgCl_2_ in mice shows a much significant increase in the tissue aspartate transaminase and alanine transaminase levels, whereas significantly reduced level of enzymes were observed in normal mice. Elevated levels of tissue aspartate transaminase and alanine transaminase enzymes point out the abnormal functions of liver, due to cellular necrosis and increased membrane permeability. Hence, increase in porosity of cell membrane act as an open channel for the passage of intracellular enzymes to adjacent cells and the circulating blood.^21^ Other reports suggest that HgCl_2_ intoxication affect the amino acid transporter site in ribosomal subunits, this will lead to gradual decrease in plasma protein level. In our findings, combined treatment with plant extracts showed a significant decline in tissue aspartate transaminase and alanine transaminase enzyme levels when compared to HgCl_2_-induced mice, similar studies highlight that treatment using herbal remedies practiced for liver injury will significantly decrease the tissue aspartate transaminase and alanine transaminase levels.^22^

Glutathione s-transferase and catalase are the former defense systems which act mutually against ROS. An iso-enzyme family of glutathione s-transferase binds to elec-trophilic compounds in conjugation with glutathione as a cofactor thereby elicits its cytoprotection against ROS.^23^ Catalase an antioxidant protective enzyme that scavenge the free radicals produced during toxicity. Increase in OH^-^production in subcellular region inactivates enzyme activity and thereby causes tissue damage by lipid peroxidation.^24^ Investigators have shown that it may due to over exploitation to scavenge the products of lipid peroxidation.^25^ Administration of MI leaf extracts prior to HgCl_2_ induction significantly reduces the toxicity by increasing the level of glutathione s-transferase and catalase enzymes. When compared with the EMI extract treatment, MMI extract elicits better scavenging property. The histomorphological consequence of HgCl_2 _treated mice shows abnormal fat cyst formation followed by nucleoli pushed to the peripheral region in liver of mice, whereas administration of EMI and MMI extracts induced with HgCl_2_ showed prominent recovery of hepatocyte. However, MMI extract exhibits maximum transformation of damaged cells into normal cells. Hence, our obtained results suggest that supplementation of MI extract prevents free radical-induced oxidative events when intoxicated with HgCl^2^

## conclusion

This study suggests that oral administration of MI extract acts as an effective radical quencher by augmenting the levels of antioxidant enzymes and guards the cells from lipid peroxidation. Tissue morphological signs also confer its protective effect against HgCl_2_ intoxication. Further experimental studies at molecular approach will be required to establish the effective action of the drug, which might be developed as a potent chemotherapeutic agent to treat liver disorders.
